# Book Reviews

**Published:** 1875-02-15

**Authors:** 


### Book Reviews.

ON THE TREATMENT OF PLEURISY, with an Appendix of Cases, etc., by John W. Carson, M. D. New York: Wm. Wood & Co. 12mo, 31 pages.

The author of this little pamphlet has had unusual opportunities for experience and observation on the treatment of pleurisy. The cases given occurred mainly among the poorer classes of foreigners and emigrants who applied for treatment at the New York and Eastern Dispensaries. The internal treatment advised by the author, does not vary materially from that ordinarily pursued. Most especial advantages are claimed, however, from the external application of a combination of croton oil, ether and iodine, as a counter-irritant. Two formulas are given—a milder croton oil paint, as follows:

| | |
|---|---|
| ℞.—Olei croton tiglii, | ʒ i. |
| Ether sulph | ʒ ii. |
| Tinct. iodine, | ʒ v. |

M. To be applied two or three coats at a time, with a camel's hair brush, over a small surface, once a week.

This is most useful for children, females, and sensitive males.

The stronger croton oil paint is given as follows:

| | |
|---|---|
| ℞.—Oleicroton tiglii, | ʒ ii. |
| Ether sulph. | ʒ iv. |
| Tinct. iodini, | ʒ ii. |
| Potass. iod., | ℈i. |
| Iodine, | grs. x. |

M. Paint as before.

These croton oil paints are advocated by the author as a substitute for blistering, in all cases where a counter-irritant effect is desired. They produce less debilitating effect, and are more steady and continuous in their effects.

EATING FOR STRENGTH: A Book Comprising 1st, The Science of Eating; 2d, Receipts for Wholesome Cookery; 3d, Receipts for Wholesome Drinks; 4th, Answers to Ever-Recurring Questions; by W. L. Holbrook, M.D. New York: Wood & Holbrook. 12mo, 156 pp.

The extended and comprehensive title given above, sufficiently explains the scope of this little work. It is designed especially for general and popular reading, its author being widely known as the editor of the *Herald of Health*, and a writer on popular hygiene and kindred topics. The views advanced and instructions given are, in the main, correct and judicious. This, and similar works treating of the general principles underlying the selection and preparation of healthy, nutritious food, should be more generally read and studied by housekeepers, wives and mothers.

---

ERYSIPELAS AND CHILD-BED FEVER, by Thos. C. Minor, M.D. Cincinnati: Robert Clarke & Co.

This treatise contains the results of the author's investigations into the connection said to exist between puerperal fever and erysipelas, including a short account of both diseases as they prevailed sporadically in the United States during the "census year, 1870," and an appendix containing the history of a puerperal fever epidemic observed in south-western Ohio, in the winter of 1872. It includes a large amount of historical and statistical material, collated from various sources, which apparently demonstrate a close connection as existing between the two diseases, erysipelas and puerperal fever, in their epidemic forms.

EXPERIMENTS ON ANIMALS AS A MEANS OF KNOWLEDGE IN PHYSIOLOGY, PATHOLOGY AND PRACTICAL MEDICINE, by J. C. Dalton, M.D. New York: F. W. Christern, 77 University Place.

This is a brief essay in defense of vivisections and experimentation upon living animals, intended to place the practice, and the calls and necessities for it, in its true and proper light before the public.

---

A GUIDE TO THE PRACTICAL EXAMINATION OF URINE, FOR THE USE OF PHYSICIANS AND STUDENTS, by James Tyson, M.D. Illustrated. Philadelphia: Lindsay & Blakiston. Chicago: Keen, Cooke & Co. 12mo, 180 pages. Price $1.50.

A plain, practical and concise little treatise on urinary analysis; it is sure to be favorably received by both physicians and students, The illustrations are numerous, and unusually well executed.

---

A SKETCH OF THE EARLY HISTORY OF PRACTICAL ANATOMY, the Introductory Address to the Course of Lectures on Anatomy at the Philadelphia School of Anatomy, by W. W. Keen, M.D. J. B. Lippincott: Philadelphia. 12mo, 40 pp.

---

PHYSICIANS' PRESCRIPTION BOOK, by Jonathan Pereira, M.D., F. R. S. Fourteenth Edition. Philadelphia: Lindsay & Blakiston.

---

OFFICE AND PRACTICE FOR SALE.

A good office and furniture for sale *very cheap*, with the succession to a practice guaranteed to be worth between two thousand five hundred and three thousand dollars per annum. Good reasons for selling. Inquire of the publishers MEDICAL EXAMINER for particulars, or address

J. W. DYSART, M.D.
Franklin Grove, Lee County, Ills.

